# The use of intravenous iron in pregnancy: for whom and when? A survey of Australian and New Zealand obstetricians

**DOI:** 10.1186/s12884-020-03363-3

**Published:** 2020-11-04

**Authors:** Sarah Smith-Wade, Giselle Kidson-Gerber, Antonia Shand, Luke Grzeskowiak, Amanda Henry

**Affiliations:** 1 School of Women’s and Children’s Health, University of New South Wales, Kensington, NSW Australia; 2 Haematology Department, Prince of Wales Hospital, Randwick, NSW Australia; 3 Children’s Hospital at Westmead Clinical School, The University of Sydney, Sydney, NSW Australia; 4 The Royal Hospital for Women, Randwick, NSW Australia; 5 Adelaide Medical School, Robinson Research Institute, University of Adelaide, Adelaide, SA Australia; 6 SA Pharmacy, Flinders Medical Centre, SA Health, Bedford Park, Adelaide, SA Australia; 7 Department of Women’s and Children’s Health, Level 2, Prichard Wing, St George Hospital, Sydney, NSW Australia; 8 The George Institute for Global Health, Sydney, NSW Australia

## Abstract

**Background:**

Iron deficiency anaemia in pregnancy (IDAP) affects 11–18% of Australian pregnancies and is associated with adverse perinatal outcomes. National prescribing data suggests the use of intravenous iron in pregnancy is increasingly common. This study aimed to: 1) Establish the current patterns of intravenous iron use by Fellows of the Royal Australian and New Zealand College of Obstetricians (FRANZCOG) when treating iron deficiency and IDAP including immediately postpartum and; 2) Assess FRANZCOG opinions regarding potential trial of intravenous iron for first-line treatment of IDAP.

**Methods:**

An online survey of RANZCOG Fellows practicing obstetrics was distributed in September 2018. Results were analysed descriptively and responses compared by clinician demographics using Chi-squared testing.

**Results:**

Of 484 respondents (21% of FRANZCOG), 457 were currently practicing obstetrics. Most prescribed intravenous iron in pregnancy (96%) and/or postpartum (85%). Most intravenous iron was prescribed for IDAP (98%) rather than iron deficiency without anaemia (53%), and for IDAP most commonly second-line to failed oral iron supplementation and first-line in special circumstances (59%). Intravenous iron prescribing was associated with shorter time since FRANZCOG completion (*p* = 0.01), public hospital practice (*p* = 0.008) and higher hospital birth numbers (p = 0.01). Most respondents (90%) would consider a randomised controlled trial of first-line intravenous iron for IDAP, although views on appropriate thresholds differed.

**Conclusions:**

Almost all respondents prescribed intravenous iron for IDAP, and while mostly used for second-line treatment over half sometimes used it first-line. With accelerating intravenous iron use, further research is required into its optimal use in pregnancy, recognizing important clinical outcomes and cost effectiveness.

**Supplementary Information:**

The online version contains supplementary material available at 10.1186/s12884-020-03363-3.

## Background

The prevalence of iron deficiency anaemia in pregnancy (IDAP) is estimated to be 20% globally [1] and 11–18% in Australia [2, 3]. All-cause severe anaemia is associated with increased maternal risks of blood transfusion, prolonged hospitalisation and maternal mortality [4, 5], alongside increased infant risks of perinatal death, small for gestational age and premature delivery [6, 7]. Given these associations the importance of treating IDAP is generally well accepted, despite limited quality data on the correlation between treatment and clinical outcomes [8].

The treatment of iron deficiency (ID) in pregnancy, with or without anaemia, has undergone significant shifts recently. This is multifactorial, including more screening and hence greater diagnosis of ID, the introduction of newer intravenous iron preparations [9] and increased recognition of the importance of patient blood management with new national guidelines [10]. Current Australian maternity guidelines recommend oral iron supplementation as first-line treatment for IDAP, as a ‘Grade B’ recommendation reflecting sufficient quality literature to guide practice in most situations. However, 10–40% of women taking oral iron experience significant gastrointestinal adverse effects, negatively impacting adherence [2]. Indeed, in a recent survey of Australian women, of those with oral iron adverse effects, 20% ceased supplements before course completion (unpublished observations). For this reason, Australian guidelines have recently been updated to recommend either lower dose or intermittent dosing of oral iron supplementation to improve tolerability.

Australian antenatal care guideline recommend intravenous iron when there is poor response to or inability to comply with oral therapy [11]. Additionally, Australian patient blood management guidelines recommend intravenous iron first-line when “rapid restoration … is required”, such as when delivery is imminent [10].

Rates of intravenous iron use more than doubled in Australian women of reproductive age between 2014 and 2017 [9, 12] which has been attributed to greater recognition of the adverse effects of oral iron and the “ease” of intravenous iron administration [13]. A recent meta-analysis suggests there may be a decreased need for blood transfusion in women treated with intravenous versus oral iron for IDAP, however the quality of the evidence was rated as low [14] with many studies of poor quality and not measuring important clinical outcomes [15]. Furthermore, intravenous iron has cost implications [12], is infrequently associated with serious adverse effects, including major allergic reactions in 3.6/1000 women [16] and carries a risk of skin staining that has led to a number of notifications to medical defence organisations in Australia [17].

The increased use of intravenous iron in Australia warrants a better understanding of current perinatal prescribing practices. This study therefore aimed to identify the current knowledge, attitudes and behaviours regarding intravenous iron prescription of fellows of the Royal Australian and New Zealand College of Obstetricians and Gynaecologists (RANZCOG) including indications for intravenous iron, frequency of prescribing, perceived advantages and disadvantages, and theoretical acceptability of a randomised trial of intravenous iron for first-line IDAP treatment.

## Methods

An anonymous survey of Fellows of the Royal Australian and New Zealand College of Obstetricians and Gynaecologists (FRANZCOG) was undertaken in September 2018. The online survey (Appendix 1) was emailed by the RANZCOG to all Fellows (*n* = 2275), using the online platform SurveyMonkey™. A follow-up reminder was sent after 2 weeks and the survey was closed after 6 weeks.

Eligible clinicians were FRANZCOGs currently working in the field of obstetrics or both obstetrics and gynaecology. FRANZCOGs who stated they were only working in gynaecology were directed to survey exit and not included in analysis. Survey completion was taken to indicate consent.

Clinician demographic questions included practice duration, region, setting and births/annum. Questions on intravenous iron prescribing focused on the setting, including respectively: a) IDAP: gestation, circumstances under which intravenous iron was prescribed, advantages and disadvantages; b) ID without anaemia in pregnancy: gestation and indication; c) Postpartum: use of intravenous iron. Thresholds for inclusion in a theoretical future randomised controlled trial (RCT) of first-line intravenous iron in pregnancy in the second or third trimester were also identified.

Statistical analysis was performed using IBM SPSS Statistics 25 (IBM SPSS Statistics 25 for Windows, IBM Corporation, Armonk, NY). Descriptive analysis of demographic characteristics included frequency and percentage tabulation while free text responses were analysed using common theme examination. Association between intravenous iron prescribing in pregnancy and clinician demographics was assessed, including time since obtaining FRANZCOG, practice area, setting and number of births. Similarly, association between clinician subgroups including practice setting and duration of FRANZCOG with IDAP and ID without anaemia treatment approaches, was assessed using either Pearsons’ Chi-Squared or Fisher’s Exact tests where appropriate.

Ethical approval was granted by the Human Research Ethics Committee of the South Eastern Sydney Local Health District (SESLHD HREC 16/371).

## Results

Overall, 484 fellows responded to the survey (response rate = 21%), of whom the majority were obstetricians and gynaecologists (*n* = 388) or obstetricians alone (*n* = 69). The small minority who practiced solely as gynaecologists (*n* = 27) were excluded from all further analyses. Most clinicians practiced in New South Wales (26%), Victoria (21%) or Queensland (19%), with the majority working in metropolitan centres (public *n =* 239, private *n =* 170) **(**Table 1**).**

**Table 1:** Clinician Demographics

	Frequency (n)	Percentage (%)
**Clinician type** (*n* = 457)		
Obstetricians	69	15.1
Both obstetricians and gynaecologists	388	84.9
**Time since obtaining FRANZCOG or overseas equivalent** (*n* = 444)		
< 5 years	104	23.4
5–9 years	74	16.7
10–19 years	115	25.9
20 years or more	148	33.3
Prefer not to say	3	0.7
**Area of practice** (n = 444)		
New South Wales	116	26.1
Victoria	91	20.5
Queensland	83	18.7
Western Australia	42	9.5
North Island New Zealand	35	7.9
South Australia	30	6.8
South Island New Zealand	19	4.3
Tasmania	10	2.3
Australian Capital Territory	8	1.8
Prefer not to say	6	1.4
Northern Territory	4	0.9
**Practice setting** (n = 444)^a^		
Metropolitan public hospital	239	53.8
Metropolitan private hospital	170	38.3
Non-metropolitan public hospital	107	24.1
Non-metropolitan private hospital	33	7.4
Prefer not to say	3	0.7
Other^b^	3	0.7
**Births per annum in largest hospital of practice** (n = 444)		
< 1000	73	16.4
1000–2499	119	26.8
2500–3999	106	23.9
4000 or more	146	32.9
**Number of women with IDAP treated per annum** (*n* = 419)		
< 10	42	10.0
10–24	108	25.8
25–49	99	23.6
≥ 50	141	33.7
Not sure	29	6.9
**Number of women with ID without anaemia treated per annum** (*n* = 413)		
< 10	70	16.9
10–24	91	22.0
25–49	65	15.7
≥ 50	136	32.9
Not sure	51	12.3

Abbreviations: *FRANZCOG* Fellowship of Royal Australian and New Zealand College of Obstetricians and Gynaecologists; *IDAP* iron-deficiency anaemia in pregnancy; ID, iron deficiency

^a^Able to select multiple practice settings

^b^Other practice settings (n = 3) included country practice, academia and private practice

### Intravenous iron prescribing

Almost all respondents indicated they prescribe intravenous iron in pregnancy (96%) and/or postpartum (85%), with administration predominantly hospital-based (92%) **(**Table 2**).** A third of clinicians prescribed fewer than 10 infusions per annum (31%), while a quarter prescribed 10–19 (25%) or 30 or more (23%) infusions per year. The most commonly prescribed formulation was ferric carboxymaltose (FCM) (90%). Intravenous iron was mostly prescribed in the third trimester, although a minority prescribed it in the first trimester (8% for IDAP, 3% for ID only).

**Table 2:** Intravenous iron prescribing practices

Question (n = number of responses)	Frequency (n)	Percentage (%)
**Prescribe IV iron in pregnancy** (n = 444)		
Yes	426	95.9
No	18	4.1
**Prescribe IV iron postpartum (***n* = 429)		
Yes	364	84.8
No	65	15.2
**Location of IV iron administration** (*n* = 421)		
Hospital	386	91.7
Non-hospital	4	1.0
Both	31	7.4
**Number of infusions prescribed in pregnancy and/or postpartum per annum** (*n* = 425)		
< 10	132	31.1
10–19	107	25.2
20–29	80	18.8
30 or more	97	22.8
Don’t prescribe	9	2.1
**IV preparation prescribed** (n = 425)^a^		
Ferric carboxymaltose (Ferinject)	381	89.6
Iron polymaltose (Ferrosig, Ferrum-H)	54	12.7
Don’t know	16	3.8
Iron sucrose (Venofer)	11	2.6
Other^a^	7	1.6
**Gestation range of IV iron prescribing in IDAP (n = 419)**^b^		
Prescribe during pregnancy	410	97.9
< 13 weeks	35	8.4
13–27 weeks	145	34.6
≥ 28 weeks	406	96.9
Do not prescribe in pregnancy	9	2.1
**Gestation range of IV iron prescribing in pregnancy for ID without anaemia (n = 413)**^**7**^		
Prescribe during pregnancy	220	53.3
< 13 weeks	14	3.4
13–27 weeks	51	12.3
≥ 28 weeks	220	53.3
Do not prescribe	193	46.7

Abbreviations: *IV* intravenous; *IDAP* iron deficiency anaemia in pregnancy; *ID* iron deficiency. Superscript: *F* Fisher’s Exact; *C* Pearson’s Chi-Squared

^a^Nil alternative infusions specified

^b^Participants could select multiple ranges

Overall differences in prescribing practices were small. Those who had obtained their FRANZCOG or overseas equivalent < 10 years ago were more likely to prescribe intravenous iron (100% vs 94% with FRANZCOG ≥10 years, *P* = 0.02), as were those practicing in public hospitals (97% vs 91% for those who were not, *P* = 0.008) and in hospitals with birth numbers > 2500/annum (98% vs 93% with < 2500/annum, *P* = 0.01) **(**Table 3**)**.

**Table 3:** Intravenous iron prescribing in pregnancy and association with obstetrician demographics

	Frequency (n) and percentage (%)	IV iron prescribing in pregnancy (n, %)^a^		***P-value***
		Yes	No	
**Time since obtaining FRANZCOG or overseas equivalent** (*n* = 441)				
< 10 years	178 (40.4)	176 (98.9)	2 (1.1)	0.014^C^*
≥ 10 years	263 (59.6)	248 (94.3)	15 (5.7)	
**Area of practice** (*n* = 438)				
Australia	384 (87.7)	368 (95.8)	16 (4.2)	0.707^F^
New Zealand	54 (12.3)	53 (98.1)	1 (1.9)	
**Practice setting (**n = 441)				
Metropolitan^b^	323 (73.2)	310 (96.0)	13 (4.0)	1.000^F^
Non-metropolitan	118 (26.8)	113 (95.8)	5 (4.2)	
Public^c^	342 (77.6)	333 (97.4)	9 (2.6)	0.008^F^**
Not public	99 (22.4)	90 (90.9)	9 (9.1)	
**Births per annum in largest hospital of practice** (n = 444)				
< 2500	192 (43.2)	179 (93.2)	13 (6.8)	0.011^C^***
> 2500	252 (56.8)	247 (98.0)	5 (2.0)	

Abbreviations: *IV* intravenous; *FRANZCOG* Fellowship of Royal Australian and New Zealand College of Obstetricians and Gynaecologists. Superscript: F, Fisher’s Exact; C, Pearson’s Chi-Squared

^a^*n* = 444

^b^“Metropolitan” represents clinicians whose practice sites include metropolitan public and/or metropolitan private hospitals; “non-metropolitan” includes clinicians who practice exclusively in non-metropolitan public hospitals, non-metropolitan private hospitals or others

^c^“Public” represents clinicians whose practice sites include metropolitan public and/or non-metropolitan public hospitals; “not public” includes clinicians who practice exclusively in metropolitan private hospitals, non-metropolitan private hospitals or others

Perceived advantages of intravenous iron in pregnancy included improvement of iron status parameters in those with poor oral iron tolerance (92%) and adherence (76%), late pregnancy IDAP or special circumstances (76%), and its rapid improvement of iron status parameters (60%). Among ‘other’ advantages specified were specific difficulties of oral iron use (*n* = 6), the avoidance of blood transfusion (*n* = 4), improvement of iron stores following postpartum haemorrhage (PPH) (*n* = 3), reduced PPH risk (*n* = 1) and maternal QOL outcomes (n = 1) **(**Table 4**).**

**Table 4:** Free text responses

Common themes	Representative responses
**Advantages of IV iron**	
Specific difficulties with oral iron (n = 6)	*Low ferritin, not responding to oral iron* *Avoid side effects of oral iron* *Noncompliance with oral iron* *Avoids the daily hassle of taking oral tabs*
Avoidance of blood transfusion (*n* = 4)	*Reduction in need for transfusion peri-partum*
	*Well timed, it can be a step to consider before packed RBCs transfusion, although this consideration has more merit in the immediate postpartum period*
	*Useful after a mild postpartum haemorrhage*
	*Postpartum haemorrhage when Hb is between 7 and 9*
Reduced PPH risk (n = 1)	*Women bleed less at delivery*
Maternal QOL outcomes (n = 1)	*Health related QOL outcomes* *https://www.ncbi.nlm.nih.gov/pmc/articles/PMC3488743/*
Others (*n* = 8)	*Although NOT in favour of infusion, hospital dictates one to implement infusion* *Symptomatic women* *You know the iron status will improve* *Severe iron deficiency*
**Disadvantages of IV iron**	
Anaphylaxis (n = 18)	*Risk of anaphylaxis (although low)* *Anaphylaxis 1/1000 = risk to mother and baby*
Other adverse effects (n = 12)	*Potential side effects* *Adverse reactions in up to 20%*
Risk of allergic reactions (n = 10)	*Possibility of allergic reactions* *Risk of allergic and anaphylactoid events not inconsiderable and overall adverse reactions - a few/100*
Administrative difficulties (*n* = 4)	*Tedious process to prescribe. Too much admin for clinic*
Cost and resource factors (n = 4)	*Cost of (hospital) admission to administer the drug* *Cost to health services and personal needed*
Over-prescription (n = 3)	*Over prescribed,* e.g. *women with low serum iron but normal Hb* *It’s often not necessary*
Lack of evidence regarding improved pregnancy outcomes (*n* = 2)	*Lack of evidence on its efficacy* *No evidence of improved outcome of pregnancy; possible increased adverse outcome*
Others (*n* = 7)	*Minimal disadvantages - a few have reactions, mainly fever but not seen major adverse effects* *Most women prefer it compared to side effects of oral iron* *Risks of toxicity, huge doses used are above physiological, risks of free radical generation*
**Indications for IV iron in the treatment of iron deficiency without anaemia**	
Symptomatic iron deficiency (*n* = 9)	*Extreme tiredness after other options tried* *Very rarely if restless legs or severe fatigue if not tolerating oral supplementation*
Special circumstances (n = 4)	*Hyperemesis with PICC (peripherally inserted central) line, influenza or bronchitis, bowel disease* *I would only do this where a woman was unable to access regular antenatal care … and where oral iron was not well tolerated.* *Intolerance to oral iron in a Jehovah’s witness with a high bleeding risk*
Not used (n = 4)	*I have recently modified my practice so that the target is Hb not ferritin. I therefore … modify oral iron supplements … and actively try to avoid IV infusions of iron* *In my institution I do not prescribe, but many others do*
Women’s preference (n = 3)	*Women prefer it. Most of the women I see dislike oral replacement and avoid secondary to sides effects,* i.e. *constipation, nausea* *Patient request*
Response to oral iron (n = 3)	*Failure of oral iron supplements with falling Hb AND altered RBC indices* *Lack of response to oral iron*
Precaution in case of PPH (n = 2)	*To increase iron store as a precaution against PPH*
Other (n = 4)	*Only on the advice of obstetric medicine colleagues* *Iron deficiency (with or without anaemia) is a condition that requires treatment.* *Aim for normal iron stores in fetus* *Would consider on case by case. Wouldn’t routinely measure iron unless anaemic or if notable drop over successive trimesters.*

Abbreviations: *IV* intravenous; *RBC* red blood cell; *PPH* postpartum haemorrhage; *Hb* haemoglobin; *QOL* quality of life

Disadvantages identified by clinicians included maternal adverse outcomes (58%), requirement for venepuncture (57%), practical difficulties of administration (44%) and high cost to the health service (31%). ‘Other’ commonly identified disadvantages included the risk of anaphylaxis (*n* = 18), risk of allergic reactions (*n* = 10) and other adverse effects (*n* = 12) **(**Table 4**).**

**Fig. 1:**
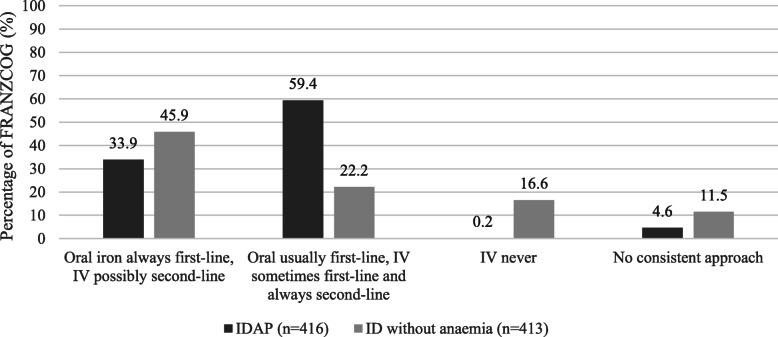
Treatment approach to iron deficiency anaemia in pregnancy (IDAP) and iron deficiency (ID) without anaemia

The principal treatment approach for IDAP was ‘oral iron usually first-line therapy, intravenous iron may be used as first-line therapy in special circumstances, and intravenous iron always used as second-line therapy if first-line oral iron fails’ (59%). For ID without anaemia, the predominant treatment approach stated by respondents was ‘oral iron always as first-line therapy, intravenous iron may be used as second-line therapy in certain circumstances (e.g. patient intolerant of oral iron and still iron deficient late in pregnancy)’ (46%) **(**Fig. 1**).** There were no differences in approach to the treatment of IDAP or ID without anaemia in pregnancy by clinicians in private practice compared to public practice (Table 5).

**Table 5:** Treatment approach in pregnancy and association with practice setting

		Frequency (n)^**a**^ and percentage ***(%)***	Practice setting (n) and percentage (%)^b**,c**^		*p-value*
			Public	Not public	
**Oral iron always first-line, IV possibly second-line**					
IDAP	*Yes*	141 (33.9)	112 (79.4)	29 (20.6)	0.705^C^
	*No*	275 (66.1)	214 (77.8)	61 (22.2)	
ID without anaemia	*Yes*	188 (45.9)	148 (78.7)	40 (21.3)	0.846^C^
	*No*	222 (54.1)	173 (77.9)	49 (22.1)	
**Oral usually first-line, IV sometimes first-line and always second-line**					
IDAP	*Yes*	247 (59.4)	192 (77.7)	55 (22.3)	0.705^C^
	*No*	169 (40.6)	134 (79.3)	35 (20.7)	
ID without anaemia	*Yes*	91 (22.2)	65 (71.4)	26 (28.5)	0.072^C^
	*No*	319 (77.8)	256 (80.3)	63 (19.7)	
**IV usually first-line**					
IDAP	*Yes*	3 (0.7)	3 (100)	0 (0)	1.000^F^
	*No*	413 (99.3)	323 (78.2)	90 (21.8)	
ID without anaemia	*Yes*	2 (0.5)	2 (100)	0 (0)	1.000^F^
	*No*	408 (99.5)	319 (78.2)	89 (21.8)	
**IV never**					
IDAP	*Yes*	1 (0.2)	1 (100))	0 (0)	1.000^F^
	*No*	415 (99.8)	325 (78.3)	90 (21.7)	
ID without anaemia	*Yes*	68 (16.6)	55 (80.9)	13 (19.1)	0.571^C^
	*No*	342 (83.4)	266 (77.8)	76 (22.2)	
**No consistent policy**					
IDAP	*Yes*	19 (4.6)	14 (73.7)	5 (26.3)	0.575^C^
	*No*	397 (95.4)	312 (78.6)	85 (21.4)	
ID without anaemia	*Yes*	47 (11.5)	38 (80.9)	9 (19.1)	0.651^C^
	*No*	363 (88.5)	283 (78.0)	80 (22.0)	

Abbreviations: *IV* intravenous; *IDAP* iron deficiency anaemia in pregnancy; *ID* iron deficiency. Superscript: F, Fisher’s Exact; C, Pearson’s Chi-Squared

^a^For IDAP *n* = 416; For ID without anaemia n = 413

^b^For practice setting analysis IDAP n = 416, ID without anaemia *n* = 410

^**c**^“Public” represents clinicians whose practice sites include metropolitan public and/or non-metropolitan public hospitals; “not public” includes clinicians who practice exclusively in metropolitan private hospitals, non-metropolitan private hospitals or others

Reasons for prescribing intravenous iron for ID without anaemia in pregnancy included intolerance of oral iron (44%), women identifying as Jehovah’s witness (32%), high bleeding risk (31%) and convenience (5%). Common theme analysis of ‘other’ reasons (*n* = 29), indicated importance of symptoms including extreme maternal fatigue **(**Table 4**)**. Forty-three percent of clinicians (*n* = 178) stated they do not prescribe intravenous iron in this context.

Common indications for the use of intravenous iron postpartum included presence of symptoms (68%), oral iron intolerance (51%), special circumstances such as Jehovah’s witness (50%), and likely oral iron nonadherence post-discharge (43%). Where clinicians indicated that they only prescribe intravenous iron postpartum below a certain haemoglobin threshold (58%), of those that specified levels these included < 70 g/L (*n* = 5), < 80 g/L (*n* = 16), < 90 g/L (*n* = 26) with one clinician additionally specifying acute bleed, < 100 g/L (*n* = 21) with additional specifiers including postpartum haemorrhage and other symptoms, < 110 g/L (n = 1) and < 115 g/L (n = 1).

### Thresholds for theoretical trial inclusion

Most clinicians (90%) would consider a theoretical randomised trial as an option. Inclusion thresholds for a theoretical RCT of first-line oral iron versus intravenous iron in pregnancy in order of acceptability were IDAP with Hb < 100 g/L (54%), IDAP with any Hb threshold in the presence of special circumstances (47%), followed by IDAP with Hb < 90 g/L (36%) and ID with Ferritin < 15 μg/L (35%) **(**Table 6**).** Identifying IDAP with any level Hb as an appropriate threshold for inclusion was associated with public practice setting (*P* = 0.04), while perceiving that the trial was not an option was associated with longer FRANZCOG duration (*P* = 0.009).

**Table 6:** Inclusion thresholds for theoretical trial and association with practice setting and FRANZCOG Duration

Thresholds deemed appropriate		Frequency (n)^**a**^ and percentage (%)	Practice setting (n)^**a**^ and percentage (%)		***p-value***	Time since obtaining FRANZCOG (n) and percentage (%)		***p-value***
			Public	Not public		< 10 years	> 10 years	
**IDAP**								
Any Hb	*Yes*	114 (26.6)	79 (69.9)	34 (30.1)	0.038^C^*	50 (43.9)	64 (56.1)	0.243^C^
	*No*	314 (73.4)	248 (79.5)	64 (20.5)		117 (37.6)	194 (62.4)	
Hb < 100 g/L	*Yes*	232 (54.2)	177 (77.0)	53 (23.0)	0.993^C^	97 (42.0)	134 (58.0)	0.214^C^
	*No*	196 (45.8)	150 (76.9)	45 (23.1)		70 (36.1)	124 (63.9)	
Hb < 90 g/L	*Yes*	155 (36.2)	116 (75.8)	37 (24.2)	0.680^C^	57 (36.8)	98 (63.2)	0.420^C^
	*No*	273 (63.8)	211 (77.6)	61 (22.2)		110 (40.7)	160 (59.3)	
Hb < 80 g/L	*Yes*	140 (32.7)	105 (76.1)	33 (23.9)	0.772^C^	51 (36.3)	89 (63.6)	0.397^C^
	*No*	288 (67.3)	222 (77.4)	65 (22.6)		116 (40.7)	169 (59.3)	
Any Hb in special circumstances^b^	*Yes*	202 (47.2)	158 (78.6)	43 (21.4)	0.440^C^	78 (38.3)	123 (61.2)	0.845^C^
	*No*	226 (52.8)	169 (75.4)	55 (24.6)		89 (39.7)	135 (60.3)	
**ID without anaemia**								
Ferritin < 30 μg/L	*Yes*	42 (9.8)	29 (69.0)	13 (31.0)	0.201^C^	20 (47.6)	22 (52.4)	0.245^C^
	*No*	386 (90.2)	298 (77.8)	85 (22.2)		147 (38.4)	236 (61.6)	
Ferritin < 15 μg/L	*Yes*	151 (35.3)	116 (76.8)	35 (23.2)	0.965^C^	55 (36.7)	95 (63.3)	0.413^C^
	*No*	277 (64.7)	211 (77.0)	63 (23.0)		112 (40.7)	163 (59.3)	
**Trial not an option**	*Yes*	45 (10.5)	34 (77.3)	10 (22.7)	0.956^C^	9 (20.9)	34 (79.1)	0.009^C^**
	*No*	383 (89.5)	293 (76.9)	88 (23.1)		158 (41.4)	224 (58.6)	
**Other**	*Yes*	20 (4.7)	16 (80.0)	4 (20.0)	1.000^F^	5 (25.0)	15 (75.0)	0.180^C^
	*No*	408 (95.3)	311 (76.8)	94 (23.2)		162 (40.0)	243 (60.0)	

Abbreviations: *FRANZCOG* Fellowship of Royal Australian and New Zealand College of Obstetricians and Gynaecologists; *IDAP* iron deficiency anaemia in pregnancy; *ID* iron deficiency; *Hb* haemoglobin. Superscript: F, Fisher’s Exact; C, Pearson’s Chi-Squared

^**a**^“Public” represents clinicians whose practice sites include metropolitan public and/or non-metropolitan public hospitals; “not public” includes clinicians who practice exclusively in metropolitan private hospitals, non-metropolitan private hospitals or others

^b^Examples of special circumstances specified included late in pregnancy, Jehovah’s witness, known prior oral iron intolerance

## Discussion

This survey is the best available representation of current practice towards treatment of ID in pregnancy by specialist obstetricians in Australia and New Zealand. Given that over three quarters of obstetricians surveyed work in public hospitals, these findings may be extrapolated to public hospitals. There are high rates of intravenous iron use by obstetricians in Australia and New Zealand for ID and IDAP, predominantly in hospital settings and most commonly in later gestation. While around half of obstetricians prescribe < 20 infusions per annum, almost a quarter prescribe 30 or more infusions a year. Interestingly, 8% of clinicians stated they prescribed intravenous iron in the first trimester for either IDAP and/or ID without anaemia, despite this being contraindicated [18].

The key point highlighted by our findings is that the prescribing of FRANZCOGs for the treatment of ID in pregnancy is not consistent with Australian antenatal care guidelines or patient blood management guidelines [1, 10, 11] There is an appropriate differentiation between IDAP and ID without anaemia in pregnancy, acknowledging the evidence of increased risk of adverse maternal and fetal outcomes with maternal anaemia [4, 5, 7]. Nonetheless, intravenous iron is commonly prescribed for women with ID without anaemia, a shift in practice that has been well recognised recently. This survey highlights that some instances of use in this cohort are established indications, for example women identifying as Jehovah’s witness or of high bleeding risk [10], however convenience (5%), poor tolerance of oral iron and severity of maternal symptoms as indications for use represent a clear departure between recommendations and practice [11]. This may be interpreted as failure to capture this shift in clinicians views’ or alternately that this reiterates the lack of quality clinical outcomes data, resulting in heterogenous practice. Irrespective, increasingly liberal use of intravenous iron may overlook the potential risk of serious, albeit uncommon side effects such as anaphylaxis and permanent skin tattooing [14], particularly in settings with less rigorous benefit data. Additionally, it must be noted that the fetal safety of intravenous iron remains unclear. It has been postulated that delivering large iron loads over a short time period in intravenous infusions, may increase the risk of iron free radicals inducing oxidative damage to vulnerable placental tissues [19, 20].

Intravenous iron unequivocally improves haematological parameters, with a recent meta-analysis finding a mean difference in maternal haemoglobin of 0.85 g/L and ferritin of 63.3μg/L [14]. However, clinical outcome data such as quality of life (QOL), breast feeding rates, preterm birth and postnatal depression was not well reported in the studies included in this meta-analysis, many of which were undertaken in low and middle income countries. The 2011 Cochrane review identified the need for assessment of clinical outcomes and effects of treatments of IDAP in large, quality randomised trials [8]. Secondary endpoints of a recent Australian RCT for IDAP comparing intravenous FCM, intravenous polymaltose (IPM) and oral iron sulphate (IS) found that higher overall serum ferritin was associated with an improvement in QOL [21]. Findings of this study are difficult to interpret given the association between QOL improvement and intravenous iron was indirect; QOL was improved with higher overall serum ferritin, which was achieved in groups of women receiving intravenous iron. Similarly, an international multi-centre RCT comparing first-line FCM and oral IS demonstrated that improved pre-delivery vitality scores and social functioning were significantly associated with FCM [22]. Both studies are biased by their open-label nature, a major limitation given QOL measures are self-reported. Additionally, the latter study was sponsored by a pharmaceutical company. Without substantial evidence of clinical superiority of intravenous iron, its use in first-line treatment of IDAP and ID highlights the importance of addressing non-evidence based treatment trends before they become common practice [9].

FCM was the most commonly used intravenous preparation, consistent with its favourable safety profile with moderate or severe ADRs occurring in the realm of 3.6/1000 for FCM versus 14.0/1000 for IPM and 7.9/1000 for IS [16]. FCM also has a shorter infusion time than IPM so may have lower administration (nursing) costs for outpatients, despite the preparation itself being more costly [14, 21]. FCM was listed on the Australian Pharmaceutical Benefits Scheme in 2014 and the New Zealand Pharmaceutical Management Agency in 2017, the former to which Seeho et al., (2018) largely attributes the rise in intravenous iron use [9]. Our findings also support the significant healthcare burden of ID with or without anaemia in pregnancy, with roughly one third of respondents treating at least fifty women per annum with IDAP (34%) and ID without anaemia (33%).

Oral iron intolerance was stated as a reason for intravenous iron prescription by some obstetricians. Although oral iron treatment strategies were not addressed in this survey, it is important to highlight the recently recognised role of ‘risk mitigation’ strategies used to improve tolerance and compliance, including reduced dose elemental iron, intermittent dosing, and avoidance of twice daily dosing [13]. The recently published British Society of Haematology guidelines outline the strategies that can be used to reduce symptoms of oral iron and lead to improve compliance and hence correction of iron deficiency anaemia [20].

The findings from our study support the acceptance of a potential RCT for firstline use of intravenous iron in pregnancy for IDAP (not ID alone) with a haemoglobin cut off of 100 g/L, where most clinicians would consider including their patients. While the lack of universal cut-offs for haematological measures of iron status in pregnancy is recognised as a research need [23], lowering inclusion thresholds could meaningfully reduce a potential IDAP study cohort in an Australian and New Zealand setting, where severe IDAP is uncommon. Indeed, Khalafallah et al’s recent Australian RCT used a haemoglobin threshold extending to ≤120 g/L, well above IDAP thresholds defined by Australian guidelines and those deemed acceptable by our cohort [21]. As such, understanding factors impacting a potential trial of first-line intravenous versus oral iron for IDAP is critical to ensuring a future study utilises appropriate and feasible inclusion thresholds and measures clinical outcomes that will meaningfully inform practice.

### Limitations of this study

Strengths of this study include that it reflects current practice by specialist obstetricians in Australia and New Zealand in both urban and metropolitan areas. Our study was limited by the low response rate, however this was comparable to similar studies of the RANZCOG membership of 19–23%, and still represents a sizeable cohort [24, 25]. Nonetheless, potential self-selection bias must be acknowledged as a limitation, particularly in light of this response rate; as with any survey, those that have greater interest or more strongly held views on a topic may be more inclined to complete the survey. Another perceived limitation may be that this survey failed to capture the views of other health care professionals involved in antenatal care, particularly general practitioners who are responsible for up to 50% of intravenous iron prescribing for women of reproductive age [12]. However, although Australian national prescribing data does not provide a breakdown on intravenous iron prescribing in pregnancy versus non-pregnant reproductive-age women, our observational experience is that general practitioners are reluctant to administer intravenous iron in pregnancy outside the hospital setting, and so likely represent a small proportion of intravenous iron prescribers in pregnancy. Obstetric physicians in tertiary centres are another important prescribing group not captured by this survey, although this speciality group has few practicing members in Australia. That being said, the rationale for restricting the survey population to FRANZCOGs alone was to establish the consensus among obstetricians regarding ID treatment, as those most likely to influence local policy and to be involved in a potential trial.

## Conclusions

Many obstetricians in Australia and New Zealand prescribe intravenous iron for IDAP, while half prescribe it for ID without anaemia. Adherence to national guidelines regarding use of intravenous iron appeared sub-optimal. Further research is required into the optimal treatment of IDAP and ID without anaemia in antenatal and postnatal settings, particularly establishing important clinical outcomes. It is therefore encouraging that a prospective RCT was acceptable to most clinicians.

## Supplementary Information

**Additional file 1.**

## Data Availability

The datasets generated and analysed during the current study are included in this published article [RANZCOG Data Spreadsheet].

## Abbreviations

IDAP: Iron deficiency anaemia in pregnancy
ID: Iron deficiency
RANZCOG: Royal Australian and New Zealand College of Obstetricians and Gynaecologists
FRANZCOG: Fellowship of the Royal Australian and New Zealand College of Obstetricians and Gynaecologists
RCT: Randomised controlled trial
FCM: Ferric carboxymaltose
PPH: Postpartum haemorrhage
QOL: Quality of life
IPM: Iron polymaltose
IS: Iron sulphate
