# Supplementary material for: The use of intravenous iron in pregnancy: for whom and when? A survey of Australian and New Zealand obstetricians
Source: BMC Pregnancy Childbirth. 2020 Nov 4;20:665. doi: 10.1186/s12884-020-03363-3 (PMC7640437; doi:10.1186/s12884-020-03363-3)
Supplement: Supplementary file 1 — Additional file 1. [file 12884_2020_3363_MOESM1_ESM.pdf]

Clinician type

* 1. What area of obstetrics and/or gynaecology do you mainly work in? (Check best answer)

- ◯ Obstetrics only
- ◯ Gynaecology only
- ◯ Both obstetrics and gynaecology

Gynaecologists only

We are targeting this survey at clinicians who practice some obstetrics, so you have been referred to the end of the survey.

Thank you very much for your participation.

Some questions about you

* 2. How long since you obtained your FRANZCOG (or if overseas-trained, your first equivalent specialist qualification)? (Check best answer)

- ◯ <5 years
- ◯ 5-9 years
- ◯ 10-19 years
- ◯ 20 years or more
- ◯ Prefer not to say

* 3. In which area of Australia/New Zealand are you currently based? (Check best answer)

- ◯ New South Wales
- ◯ ACT
- ◯ Victoria
- ◯ Queensland
- ◯ South Australia
- ◯ Western Australia
- ◯ Tasmania
- ◯ Northern Territory
- ◯ North Island New Zealand
- ◯ South Island New Zealand
- ◯ Prefer not to say

* 4. In what setting(s) do you provide maternity care? (Check all that apply)

- ☐ Metropolitan public hospital
- ☐ Metropolitan private hospital
- ☐ Non-metropolitan public hospital
- ☐ Non-metropolitan private hospital
- ☐ Prefer not to say
- ☐ Other (please specify)

* 5. How many births per annum are there in the *largest* hospital you work at? (Check best answer)

- ◯ <1000 births/annum
- ◯ 1000-2499 births/annum
- ◯ 2500-3999 births/annum
- ◯ 4000 or more births/annum

Clinician prescribing characteristics

* 6. Have you ever prescribed intravenous (IV) iron in pregnancy?

- ◯ Yes
- ◯ No

Regarding IV iron in pregnancy

The following questions relate to the use of IV iron in pregnancy

* 7. Where do you administer iron infusions in pregnancy? (Check best answer)

- ◯ Hospital e.g. day stay, inpatient wards, outpatient clinics
- ◯ Non-hospital e.g. consulting rooms
- ◯ Both hospital and non-hospital

* 8. What do you think are the main advantages of IV iron in pregnancy? (Check all that apply)

- ☐ Rapid improvement of iron status parameters
- ☐ Improvement in iron/anaemia in women who don't tolerate oral iron
- ☐ Improvement in iron/anaemia in women with poor adherence to oral iron
- ☐ Useful for late pregnancy anaemia/special circumstances where oral insufficient
- ☐ Women prefer it
- ☐ Other (please specify)

* 9. What do you think are the main disadvantages of IV iron in pregnancy? (Check all that apply)

- ☐ Requires venepuncture
- ☐ Insufficient fetal safety data for use in pregnancy
- ☐ Practical difficulties of IV administration/time it takes etc
- ☐ High cost of medication to the patient
- ☐ High cost of medication to the health service
- ☐ Maternal adverse outcomes risk e.g. tattooing from extravasation
- ☐ Women would rather avoid IV treatment
- ☐ No disadvantages
- ☐ Other (please specify)

Regarding iron deficiency with anaemia in pregnancy

The following questions relate to the treatment of iron deficiency with anaemia in pregnancy

* 10. What is the treatment of **iron deficiency anaemia** in pregnancy in your practice/institution? (Check best answer)

- ◯ Oral iron always as first line therapy, IV iron may be used as second-line therapy in certain circumstances (e.g. patient intolerant of oral iron and still anaemic late in pregnancy)
- ◯ Oral iron usually first-line therapy, IV iron may be used as first-line therapy in special circumstances (e.g. late in pregnancy, severe anaemia, known intolerance of oral iron), and IV iron always used as second-line therapy if first-line oral iron fails
- ◯ IV iron usually first-line therapy
- ◯ IV iron is never used for this indication in my institution/practice
- ◯ No consistent policy in my institution/practice of which I am aware
- ◯ Other (please specify)

* 11. At which gestations do you use IV iron for the treatment of **iron deficiency anaemia** in pregnancy? (Check all that apply)

- ☐ Less than 13 weeks
- ☐ 13-27 weeks
- ☐ 28 weeks onwards
- ☐ I do not use IV iron for iron deficiency anaemia

* 12. How many women **with iron deficiency anaemia** in pregnancy do you manage every year, either personally or as part of a team? (Check best answer)

- ◯ Less than 10
- ◯ 10-24
- ◯ 25-49
- ◯ 50 or more
- ◯ Not sure

Regarding iron deficiency *without anaemia* in pregnancy

The following questions relate to the treatment of iron deficiency *without anaemia* in pregnancy

* 13. What is the treatment of **iron deficiency *without anaemia*** in pregnancy in your practice/institution? (Check best answer)

- ◯ Oral iron always as first line therapy, IV iron may be used as second-line therapy in certain circumstances (e.g. patient intolerant of oral iron and still iron deficient late in pregnancy)
- ◯ Oral iron usually first-line therapy, IV iron may be used as first-line therapy in special circumstances (e.g. late in pregnancy, severe anaemia, known intolerance of oral iron), and IV iron always used as second-line therapy if first-line oral iron fails
- ◯ IV iron usually first-line therapy
- ◯ IV iron is never used for this indication in my institution/practice
- ◯ No consistent policy in my institution/practice of which I am aware
- ◯ Other (please specify)

* 14. At which gestations do you use IV iron for the treatment of **iron deficiency *without anaemia*** in pregnancy? (Check all that apply)

- ☐ Less than 13 weeks
- ☐ 13-27 weeks
- ☐ 28 weeks onwards
- ☐ I do not use IV iron for iron deficiency *without anaemia* in pregnancy

* 15. If you prescribe IV iron for **iron deficiency *without anaemia*** in pregnancy, what are the main reasons? (Check all that apply)

- ☐ Intolerance of oral iron
- ☐ High bleeding risk
- ☐ Jehovah's witness
- ☐ Convenience
- ☐ I do not prescribe IV iron in this context
- ☐ Other (please specify)

* 16. How many women **with iron deficiency *without anaemia*** in pregnancy do you manage every year, either personally or as part of a team? (Check best answer)

- ◯ Less than 10
- ◯ 10-24
- ◯ 25-49
- ◯ 50 or more
- ◯ Not sure

A possible trial

We are considering a trial of IV iron in pregnancy in the second or third trimester for first-line therapy. We are interested in your views about who you would consider suitable for a trial.

* 17. Who would you consider enrolling in a trial of IV iron? (Check all that apply)

- ☐ Any woman with **iron deficiency anaemia**, *regardless of haemoglobin*
- ☐ Any woman with **iron deficiency anaemia** with a *haemoglobin <100g/L*
- ☐ Any woman with **iron deficiency anaemia** with a *haemoglobin <90g/L*
- ☐ Any woman with **iron deficiency anaemia** with a *haemoglobin <80g/L*
- ☐ Any woman with **iron deficiency anaemia**, *regardless of haemoglobin in special circumstances* (e.g. late in pregnancy, multiple pregnancy, Jehovah's witness, known prior oral intolerance)
- ☐ Any woman with **iron deficiency *without anaemia*,** with a *ferritin <30µg/L*
- ☐ Any woman with **iron deficiency *without anaemia***, with a *ferritin <15µg/L*
- ☐ I would not consider this trial an option
- ☐ Other (please specify)

Regarding postpartum iron deficiency anaemia

The following question regards the treatment of iron deficiency anaemia in the postpartum period

* 18. Do you ever use IV iron in the immediate postpartum setting (birth to hospital discharge) in your practice/institution?

- ◯ Yes
- ◯ No

Regarding use of intravenous iron postpartum

The following question regards the use of IV iron for the treatment of iron deficiency anaemia in the postpartum period

* 19. In what situations do you use IV iron in the immediate postpartum setting (birth to hospital discharge) in your practice/institution - assuming the patient is haemodynamically stable and her bleeding is no longer excessive? Check all that apply.

- [ ] If patient does not tolerate oral iron
- [ ] If patient is likely to be nonadherent with oral iron after discharge
- [ ] If patient is symptomatic
- [ ] In special circumstances (e.g. Jehovah's witness)
- [ ] Haemoglobin below a certain threshold (please specify in g/L)

Regarding intravenous iron in pregnancy and/or postpartum

The following questions regard the treatment of iron deficiency anaemia in pregnancy and/or the postpartum period

* 20. Which preparation(s) of IV iron do you prescribe? (Check all that apply)

- [ ] Iron sucrose (Venofer)
- [ ] Ferric carboxymaltose (Ferinject)
- [ ] Iron polymaltose (Ferrosig, Ferrum-H)
- [ ] Don't know
- [ ] I don't ever prescribe IV iron in pregnancy or postpartum
- [ ] Other (please specify)

* 21. How many IV iron infusions do you prescribe per year, either directly or as the consultant of the prescribing clinical team? (Check best answer)

- ○ Less than 10
- ○ 10-19
- ○ 20-29
- ○ 30 or more
- ○ I don't ever prescribe IV iron in pregnancy or postpartum
